# Clinicopathological and prognostic significance of heat shock protein 27 (HSP27) expression in non-small cell lung cancer: a systematic review and meta-analysis

**DOI:** 10.1186/s40064-016-2827-8

**Published:** 2016-07-25

**Authors:** Shuangjiang Li, Wenbiao Zhang, Jun Fan, Yutian Lai, Guowei Che

**Affiliations:** 1 Department of General Thoracic Surgery, West China Hospital, Sichuan University, Guoxue Alley No. 37, Chengdu, 610041 China; 2 Lung Cancer Center, West China Hospital, Sichuan University, Chengdu, 610041 China

**Keywords:** Heat shock protein 27, Non-small cell lung cancer, Prognosis, Meta-analysis

## Abstract

Numbers of clinical and experimental investigations have provided increasing evidences to demonstrate that heat shock protein 27 (HSP27) is a qualified predictor for many cancers. However, no consensus has been reached on its clinicopathological and prognostic significance in patients with non-small cell lung cancer (NSCLC). Therefore, we performed this systematic meta-analysis to help addressing this issue. PubMed, EMBASE, the Web of Science and China National Knowledge Infrastructure were searched for full-text literatures met out eligibility criteria. We determined the odds ratio (OR) and hazard ratio (HR) as the appropriate summarized statistics for assessments of clinicopathological and prognostic roles of HSP27, respectively. Q-test and I^2^-statistic were used to evaluate the level of heterogeneity. Sensitivity analysis was conducted to examine the stability of overall estimates. Potential publication bias was detected by Begg’s test and Egger’s test. Finally, ten articles were identified to be included into our meta-analysis. The pooled analyses suggested that HSP27 expression was significantly associated with the unfavorable conditions for differentiation degree, lymphatic metastasis, clinical stage, squamous cell carcinoma and tumor size. However, HSP27 expression had no significant relationship to gender, age and smoking status. Meanwhile, pooled HRs indicated that HSP27 expression could be a predictor for a lower 5-year overall survival (OS) rate (HR: 1.832; 95 % CI 1.322–2.538; *P* < 0.001) but not for 1-year OS of NSCLC (HR: 0.885; 95 % CI 0.140–5.599; *P* = 0.896). In conclusion, our meta-analysis demonstrates that HSP27 expression may be a strong biomarker to predict both the poor clinicopathological and prognostic characteristics in patients with NSCLC.

**Electronic supplementary material:**

The online version of this article (doi:10.1186/s40064-016-2827-8) contains supplementary material, which is available to authorized users.

## Background

Lung cancer is the leading cause of malignancy-related death around the world. Non-small cell lung cancer (NSCLC) accounts for more than 85 % of all lung cancer cases and its five-year overall survival (OS) rate approximates 15 % in both developed and developing countries (Jemal et al. 2011; Alberg et al. 2013; Efird et al. 2014). The morbidity and mortality rates have dramatically increased in both male and female patients with lung cancer during the last decade (Hoffman et al. 2000; Ferlay et al. 2010; Wu et al. 2013). According to WHO’s estimations, China will become one of the countries with relatively higher prevalence of NSCLC in this century. Advanced pathological staging, early metastasis and poor response to treatments are the potential leading causes for a poor prognosis of NSCLC. To improve clinicians’ decisions on appropriate therapeutic regimens and patient managements, it has been increasingly urgent to identify strong biomarkers that can accurately predict the clinicopathological characteristics and prognostic outcomes of NSCLC.

A great number of clinical and experimental researches have focused on the prognostic factors in NSCLC and identified many possible biomarkers. Heat shock proteins (HSPs) are a group of highly conserved proteins with essential hemostatic functions under physiological conditions and protective functions under stressful conditions (Haslbeck et al. 2005). Heat shock protein 27 (HSP27) encoded by HSPB1 is an important representative of the HSP family (Guo et al. 2010). Previous investigations have provided the increasing evidences indicating that HSP27 can be an effective predictor for many diseases, including renal injury and fibrosis, cancers, neurological degeneration and cardiovascular diseases (Vidyasagar et al. 2012). Its prognostic roles have been demonstrated in some solid tumors such as head-cervical carcinomas and colorectal cancers (Norton et al. 2010; Kaigorodova and Bogatyuk 2014). Although there have been studies attempted to clarify the clinicopathological and prognostic significances of HSP27 in NSCLC, some controversial and even totally contradictory results have appeared, resulting in that no consensus has been reached until now (Vidyasagar et al. 2012; Kaigorodova and Bogatyuk 2014).

Therefore, we performed the current systematic review and meta-analysis to help addressing this issue. First, we aim to evaluate the relationship between HSP27 expression and major clinicopathological characteristics of NSCLC. Second, we investigated the prognostic value of HSP27 on survival outcomes in patients with NSCLC. Based on applying the evidence-based methods to a larger number of enrolled samples, this meta-analysis may help to give the clinicians some suggestions to improve the prognosis of NSCLC.

## Methods

### Protocol

No protocol had been previously published for this review. A meta-analysis does not require necessary patients’ consent or ethical approval. We performed this meta-analysis in accordance with the Preferred Reporting Items for Systematic Reviews and Meta-Analyses (PRISMA) statement (Moher et al. 2009). An additional PRISMA checklist is given in the Additional file 1.

### Search strategies

No language or publication data restriction was imposed within this meta-analysis. Four universal electronic databases, including PubMed, EMBASE (via Ovid interface), the Web of Science (via campus network of Sichuan University) and China National Knowledge Infrastructure (CNKI), were selected for identifications of eligible literatures. Three search strings were combined with several key words and Boolean Operators (“AND” and “OR”). These key words involved the “heat shock protein 27”, “heat shock protein B1”, “HSP27”, “HSPB1”, “lung cancer”, “lung carcinoma” and “lung neoplasm”. The complete searching details were outlined in the Additional file 2. We also manually searched the reference lists of relevant literatures to identify any one possibly included study with no duplication.

### Inclusion and exclusion criteria

The following eligibility criteria was established to determine the eligible studies included into our meta-analysis.

*Inclusion criteria:* (I) the target disease is NSCLC, not involving small cell lung cancer (SCLC) or other lung malignances; (II) positive expression of HSP27 is independently detected, not in accompany with other biomarkers; (III) demographic details and Kaplan–Meier (K–M) survival curves assessing the prognostic roles of HSP27 and its relationship to major clinicopathological features of NSCLC are available in the original articles, and the endpoint prefers to be the OS; (IV) outcome statistics indicating the prognostic significance of HSP27, including the odds ratio (OR), hazard ratio (HR) and relative risk (RR), are directly reported in the original articles.

*Exclusion criteria:* (I) the following literature styles should be immediately excluded, including reviews, letters, laboratorial experiments and conference abstracts; (II) valid data correlated with the HSP27 expression in NSCLC were not reported; (III) the comparisons of HSP27 expression between carcinomatous tissues and normal tissues of the lung are not considered.

### Quality assessment

Newcastle-Ottawa Scale (NOS) was applied to evaluate the quality of original non-randomized studies (Stang 2010). Three perspectives including selection, comparability and exposure were considered for estimations of the quality and potential bias risks. The “star system” with a maximum of 9 stars was employed as the assessment tool. After grading all of the included studies, we regarded 8–9 stars as a good quality, 6–7 stars as a fair quality, and lower than 6 stars as a poor quality.

### Data collection

We designed an Microsoft Office Excel spreadsheet to record the following details: (I) publication data including authors, publication year and nations; (II) experimental data including study design, study period, investigating fields, detecting materials and methods, cut-off values and follow-ups; (III) demographic data including enrolled samples, the number of patients with positive expression and negative expression of HSP27; (IV) statistical data including statistical methods, outcome statistics with the corresponding 95 % confidence interval (95 % CI) and their sources, including those extrapolated by demographic and survival data or just directly reported from the original articles.

### Statistical analysis

To evaluate the association between HSP27 expression and clinicopathological characteristics in patients with NSCLC, we determined to adopt the OR with 95 % CI as the appropriate summarized statistics. If HR or RR derived from multivariate analysis was reported, we could immediately incorporate it into the meta-analysis (Li et al. 2016b).

To assess the prognostic value of HSP27 expression in patients with NSCLC, HR with 95 % CI was determined to serve as the summarized estimates because that HR was generally regarded as the only statistical value compatible for both censoring and time-to-events (Parmar et al. 1998). It would be an optimal way to incorporate the multivariate HR outcomes into the meta-analysis because that multivariate analysis using logistic regression or Cox proportional hazards model was generally used to eliminate the bias risks from other confounding factors in observational studies. If no multivariate statistic was reported, we could extrapolate univariate HR with 95 % CI from the survival data according to a practical method described by Tierney et al. (2007). The relevant formulas are given as follows:

$$ O{-}E = \frac{{\sqrt {Total\; observed\; events \times Analyzed\; research \times Analyzed \;control} }}{{\left( {Analyzed\;research + Analyzed \;control} \right)}} \times \left( {Z\; score\; for\; P\; value/2} \right) $$

$$ V = \frac{Total\;observed\;events \times Analyzed\;research \times Analyzed\;control}{{\left( {Analyzed\;research + Analyzed\;control} \right)^{2} }} $$

$$ HR = Exp\left( { - \frac{O - E}{V}} \right) $$

where O−E is the *log rank Observed minus Expected events* and V is the *log rank Variance* (Tierney et al. 2007). Then, we could extract the survival data by Engauge Digitizer 4.1 (http://sourceforge.net) from the K–M curves to measure the accuracy of estimated HR. In addition, the multivariate RR and OR could be considered as HR and incorporated into our meta-analysis (Li et al. 2016b).

Both Cochrane Q-test and I^2^-statistic were applied to estimate the level of heterogeneity within this meta-analysis. Fine heterogeneity was defined as I^2^ < 50 % and *P* > 0.1, and a fixed-effect model was employed at the same time. On the contrary, a random-effect model would be selected if a significant heterogeneity was revealed by I^2^ ≥ 50 % or *P* ≤ 0.1 (Higgins and Thompson 2002).

Sensitivity analysis was also conducted to examine the stability of integrated estimates by omitting the individual study sequentially. The strong robustness of our meta-analysis would be confirmed if there were no substantial variation between the adjusted summarized statistics and primary summarized statistics (Higgins et al. 2003).

Potential publication bias was detected by both Begg’s test and Egger’s test. Its presence was suggested by the symmetry of funnel plot conducted by Begg’s test, and in which log ORs and log HRs were plotted against their corresponding standard errors (SEs) (Begg and Mazumdar 1994). The significant bias was also suggested by Egger’s *P* value <0.05.

Finally, we declared that all of the above statistical analyses were accomplished by STATA 12.0 (STATA Corporation, College Station, TX).

## Results

### The selection of included studies

**Fig. 1:**
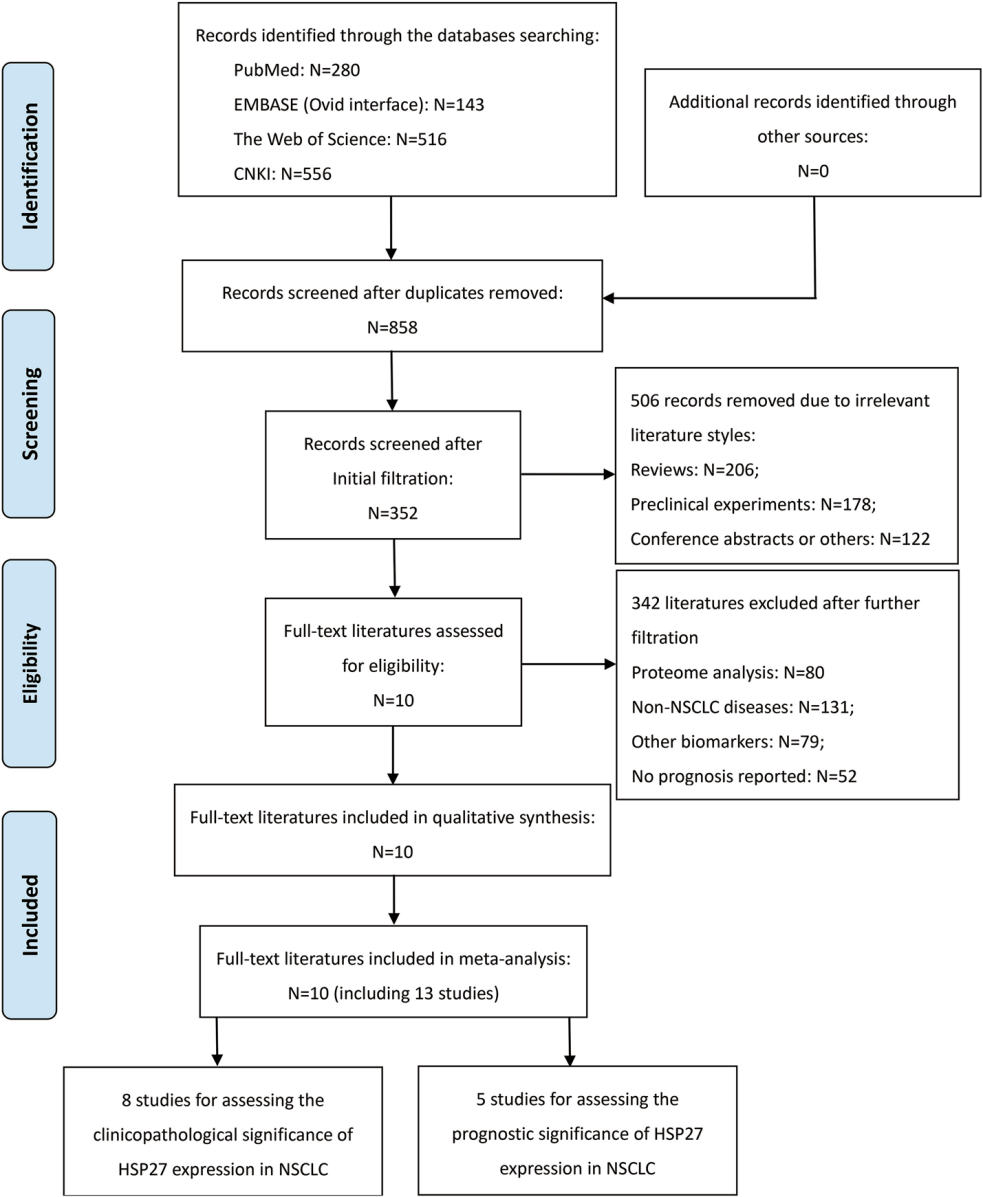
PRISMA flow diagram of the literature retrieval. HSP27, heat shock protein 27; NSCLC, non-small cell lung cancer; PRISMA, Preferred Reporting Items for Systematic Reviews and Meta-Analyses

There were a total of 1495 publication items identified by searching through the four electronic databases, including 280 citations in PubMed, 143 citations in EMBASE, 516 citations in the Web of Science and 556 citations in CNKI. There were 858 of them entered into the initial filtration after excluding the duplicated ones. The initial filtration was based on screening the titles and abstracts, while further filtration was conducted by reading through the full-text of remaining literatures. Then, ten full-text articles were identified for the possible eligibility in this meta-analysis (Huang et al. 2005; Liu et al. 2016; Malusecka et al. 2008; Tsai et al. 2014; Wang et al. 2011; Yao et al. 2009; Zhao et al. 2014a). Finally, all of these 10 articles were determined to be included into our meta-analysis, including seven English papers (Huang et al. 2005; Liu et al. 2016; Malusecka et al. 2008; Tsai et al. 2014; Wang et al. 2011; Yao et al. 2009; Zhao et al. 2014a) and three Chinese papers (Li et al. 2005; Zhao et al. 2009; Zu and Huang 2005). The main procedures and relevant details of above retrievals were shown as a PRISMA diagram (Fig. 1).

### The quality level of included studies

The quality level of each study was estimated by a NOS score, then listed by the number of stars (Table 1). Finally, their mean score was 7.4 (ranged from 6 to 8), suggesting a fairly good quality level of all the included studies.

**Table 1:** Baseline characteristics of the included studies

Authors (year)	Nation/region	Study design	Study period	NOS	No. Samples			Materials	Investigating fields		Follow-up (maximum)
					Total	PE	NE		CP features	Prognosis	
Huang et al. (2005)	China	ROS	NI	7	60	43	17	Paraffin-embedded tissue	**✓**	**✗**	–
Liu et al. (2016)	Taiwan	ROS	2009–2010	8	22	8	14	Paraffin-embedded tissue	**✓**	**✓**	60 months
Malusecka et al. (2008)	Poland	ROS	1993–1995	8	76	59	17	Paraffin-embedded tissue	**✗**	**✓**	12 months
Tsai et al. (2014)	Taiwan	ROS	2004–2010	8	64	39	25	Frozen tissue	**✗**	**✓**	12 months
Wang et al. (2011)	China	ROS	2003–2004	8	103	61	42	Paraffin-embedded tissue	**✓**	**✓**	60 months
Yao et al. (2009)	China	ROS	NI	8	147	93	54	Frozen tissue	**✓**	**✗**	–
Zhao et al. (2014a, 2014b)	China	ROS	2005	8	209	116	93	Paraffin-embedded tissue	**✓**	**✓**	66 months
Li et al. (2005)	China	ROS	2004	7	50	22	28	Paraffin-embedded tissue	**✓**	**✗**	–
Zhao et al. (2009)	China	ROS	2002–2004	6	63	40	23	Paraffin-embedded tissue	**✓**	**✗**	–
Zu and Huang (2005)	China	ROS	2002–2003	6	60	43	17	Paraffin-embedded tissue	**✓**	**✗**	–

Authors (year)	Stages	Histology			Detecting method	Cut-off value	Positive site	Estimates	Extractions	Statistical analysis
		SCC	AC	Others						
Huang et al. (2005)	I–IV	35	25	**–**	IHC	10 % staining	Cytoplasm	OR	DDE	Univariate
Liu et al. (2016)	I–IV	**–**	22	**–**	IHC	10 % staining	NI	OR, HR	DDE	Univariate
Malusecka et al. (2008)	I–III	Not mentioned			IHC	10 % staining	Cytoplasm	HR	Reported	Multivariate
Tsai et al. (2014)	I–IV	47	17	**–**	RT–PCR	Ratio = 1*	NI	HR	Reported	Multivariate
Wang et al. (2011)	I–III	**–**	103	**–**	IHC	5 % staining	Nucleus	OR, HR	DDE	Univariate
Yao et al. (2009)	I–IV	147	**–**	**–**	IHC	25 % staining	NI	OR	DDE	Univariate
Zhao et al. (2014a, 2014b)	I–IV	86	110	13	IHC	40 % staining	Cytoplasm	OR, HR	DDE	Univariate
Li et al. (2005)	I–IV	19	25	6	IHC	5 % staining	Cytoplasm	OR	DDE	Univariate
Zhao et al. (2009)	NI	Not mentioned			IHC	5 % staining	Cytoplasm	OR	DDE	Univariate
Zu and Huang (2005)	NI	35	25	**–**	IHC	10 % staining	Cytoplasm	OR	DDE	Univariate

* The ratio of HSP27 expression in carcinomatous tissues compared with its expression in adjacent normal tissues. High expression was defined by the ratio >1, and low expression was defined by the ratio <1

*AC* adenocarcinoma, *CP* clinicopathological, *DDE* demographic data extrapolated, *HR* hazard ratio, *IHC* immunohistochemistry, *NE* negative expression, *NI* no information, *NOS* Newcastle-Ottawa Scale, *OR* odds ratio, *PE* positive expression, *ROS* retrospective observational study, *SCC* squamous cell carcinoma

### The characteristics of included studies

Baseline characteristics for these ten eligible articles were summarized in Table 1. Actually, there were 13 retrospective observational studies reported from these 10 papers, including 8 of them assessing the relationship between HSP27 expression and clinicopathological features of NSCLC (Huang et al. 2005; Liu et al. 2016; Wang et al. 2011; Yao et al. 2009; Zhao et al. 2009, 2014a; Li et al. 2005; Zu and Huang 2005) and 5 of them exploring the prognostic significance of HSP27 expression for OS of NSCLC patients (Liu et al. 2016; Malusecka et al. 2008; Tsai et al. 2014; Wang et al. 2011; Zhao et al. 2014a). Totally, 854 patients diagnosed with NSCLC were enrolled from the 13 included studies, and the sample size ranged from 22 to 209. Most researchers prepared paraffin-embedded tissues as the experimental materials (Huang et al. 2005; Wang et al. 2011; Zhao et al. 2014a; Malusecka et al. 2008; Liu et al. 2016; Li et al. 2005; Zhao et al. 2009; Zu and Huang 2005), except the remaining two studies used the frozen tissues for further analysis (Tsai et al. 2014; Yao et al. 2009). Immunohistochemistry (IHC) was applied for the detection of positive expression of HSP27 among 12 studies except only one using the quantitative RT-PCR (Tsai et al. 2014). However, the positive-staining sites of HSP27 and their corresponding cut-off definitions varied across different studies (Table 1). On the basis of such concerns, the positive expression rate of HSP27 was 61.4 % (524/854) in all of the NSCLC cases.

None of all the included studies supplied any statistic estimating the association between HSP27 expression and clinicopathological characteristics of NSCLC. However, valid demographics with the corresponding *P* value were reported within all of these studies. As for the prognostic value of HSP27, two studies directly reported the multivariate HR outcomes for 1-year OS (Malusecka et al. 2008; Tsai et al. 2014) and the other three studies just provided the survival data with log-rank *P* value to assess the prognostic value of HSP27 for 5-year OS in NSCLC (Liu et al. 2016; Wang et al. 2011; Zhao et al. 2014a). In addition, the other statistical details of these ten eligible articles, including statistical analysis methods, outcome statistics and their extractions, were also summarized in Table 1.

### Association between HSP27 expression and clinicopathological characteristics of NSCLC

**Fig. 2:**
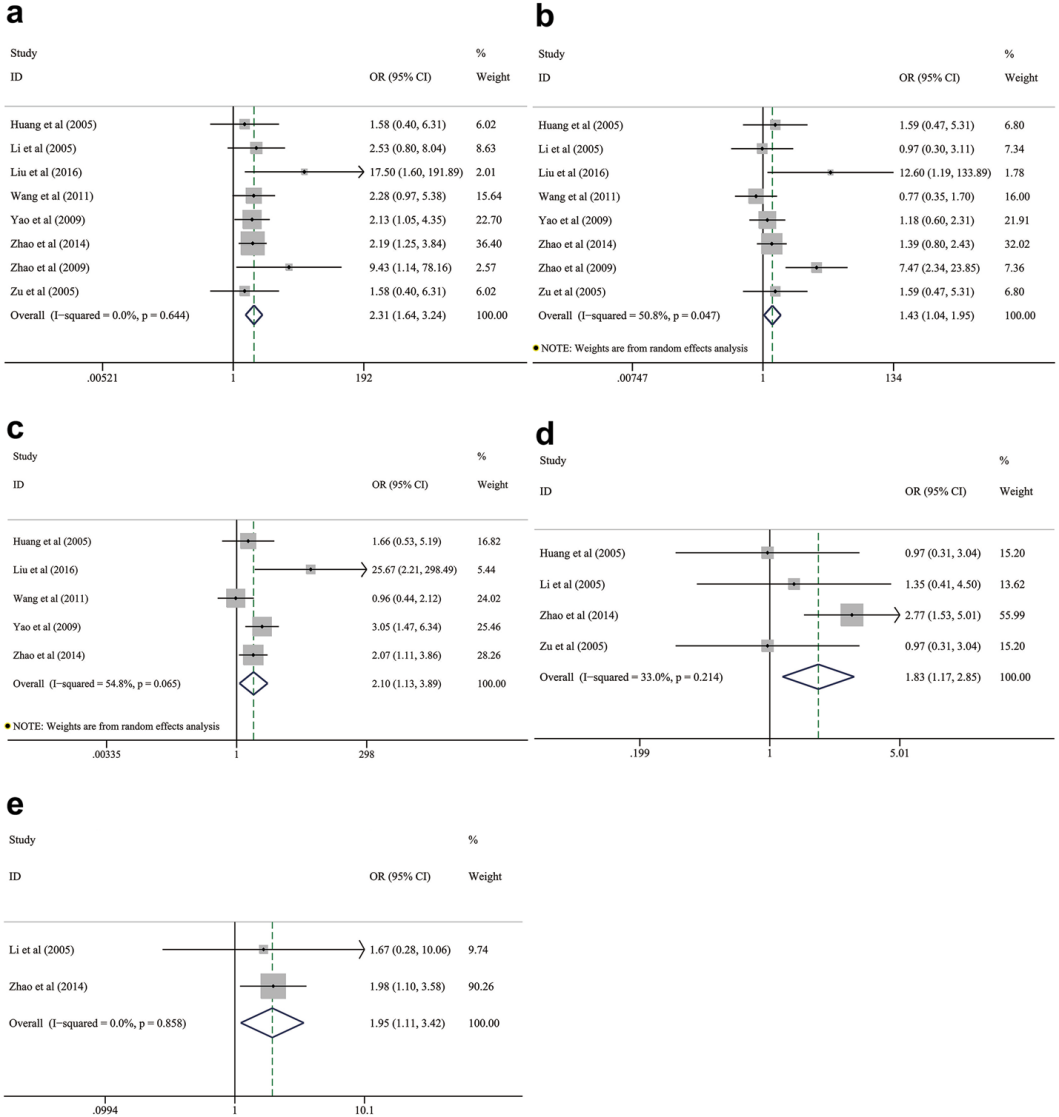
Pooled analyses for assessing the associations between HSP27 expression and **a** differentiation degree, **b** lymphatic metastasis, **c** TNM stage, **d** histological subtypes and **e** tumor size of NSCLC. *HSP27* heat shock protein 27, *NSCLC* non-small cell lung cancer

**Fig. 3:**
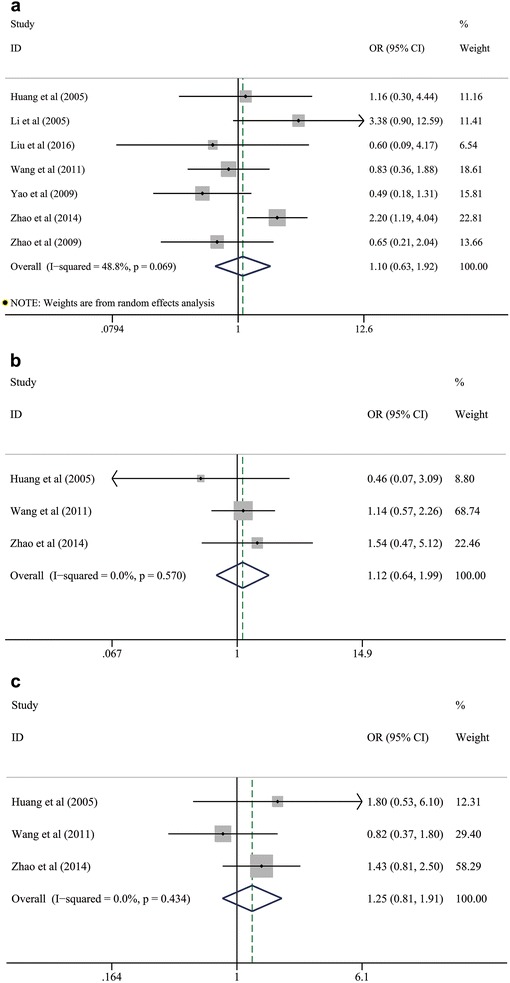
Pooled analyses for assessing the associations between HSP27 expression and **a** gender, **b** age, and **c** smoking status in patients with NSCLC. *HSP27* heat shock protein 27, *NSCLC* non-small cell lung cancer

We collected the demographic data from each included study to extrapolate the corresponding OR with 95 % CI revealing the frequency of HSP27 expression on different clinicopathological variables. These parameters involved genders, ages, smoking status, differentiation degrees, histological subtypes, TNM stages, lymphatic metastasis and tumor size. By pooling these ORs with 95 % CI of each clinicopathological variable, we found that positive expression of HSP27 was significantly associated with the lower differentiation degree (OR 2.309; 95 % CI 1.645–3.242; *P* < 0.001; I^2^ = 0.0 %, *p* = 0.644; Fig. 2a; Table 2), lymphatic metastasis (OR 1.425; 95 % CI 1.040–1.953; *P* = 0.027; I^2^ = 50.8 %, *p* = 0.047; Fig. 2b; Table 2), advanced TNM stage (OR 2.10; 95 % CI 1.134–3.888; *P* = 0.018; I^2^ = 54.8 %, *p* = 0.065; Fig. 2c; Table 2), squamous cell carcinoma (SCC) (OR 1.827; 95 % CI 1.171–2.849; *P* = 0.008; I^2^ = 33.0 %, *p* = 0.214; Fig. 2d; Table 2) and larger tumor size (OR 1.949; 95 % CI 1.112–3.416; *P* = 0.020; I^2^ = 0.0 %, *p* = 0.858; Fig. 2e; Table 2). Thus, positive expression of HSP27 seems to be significantly correlated with the worse pathological characteristics of NSCLC. However, the summarized outcomes indicated that HSP27 expression had no significant relationships with the clinical characteristics including gender (OR 1.099; 95 % CI 0.629–1.920; *P* = 0.740; I^2^ = 48.8 %, *p* = 0.069; Fig. 3a; Table 2), age (OR 1.125; 95 % CI 0.637–1.986; *P* = 0.685; I^2^ = 0.0 %, *p* = 0.570; Fig. 3b; Table 2) and smoking status (OR 1.246; 95 % CI 0.812–1.913; *P* = 0.314; I^2^ = 0.0 %, *p* = 0.434; Fig. 3c; Table 2).

**Table 2:** Meta-analysis of the clinicopathological significance of HSP27 expression in patients with NSCLC

Clinicopathological characteristics	*N*	No. samples			Heterogeneity (I-squared, *p*)	Model	OR with 95 % CI	*P* value	Conclusion
		Total	PE	NE					
Gender (male vs female)	7	654	383	271	48.8 %, 0.069	Random	1.099 (0.629–1.920)	0.740	Not significant
Age (≥60 years vs <60 years)	3	372	220	152	0.0 %, 0.570	Fixed	1.125 (0.637–1.986)	0.685	Not significant
Smoking status (yes vs no)	3	372	220	152	0.0 %, 0.434	Fixed	1.246 (0.812–1.913)	0.314	Not significant
Differentiation (G3 vs G1 and G2)	8	714	426	288	0.0 %, 0.644	Fixed	2.309 (1.645–3.242)	<0.001	Significant
Lymphatic metastasis (yes vs no)	8	714	426	288	50.8 %, 0.047	Random	1.425 (1.040–1.953)	0.027	Significant
TNM stage (III/IV vs I/II)	5	541	321	220	54.8 %, 0.065	Random	2.10 (1.134–3.888)	0.018	Significant
Histological types (SCC vs AC)	4	379	224	155	33.0 %, 0.214	Fixed	1.827 (1.171–2.849)	0.008	Significant
Tumor size (≥3 cm vs <3 cm)	2	259	138	121	0.0 %, 0.858	Fixed	1.949 (1.112–3.416)	0.020	Significant

*AC* adenocarcinoma, *CI* confidence interval, *HSP27* heat shock protein 27, *N* reference count, *NE* negative expression, *NSCLC* non-small cell lung cancer, *OR* odds ratio, *PE* positive expression, *SCC* squamous cell carcinoma

### Prognostic value of HSP27 expression for OS in patients with NSCLC

**Fig. 4:**
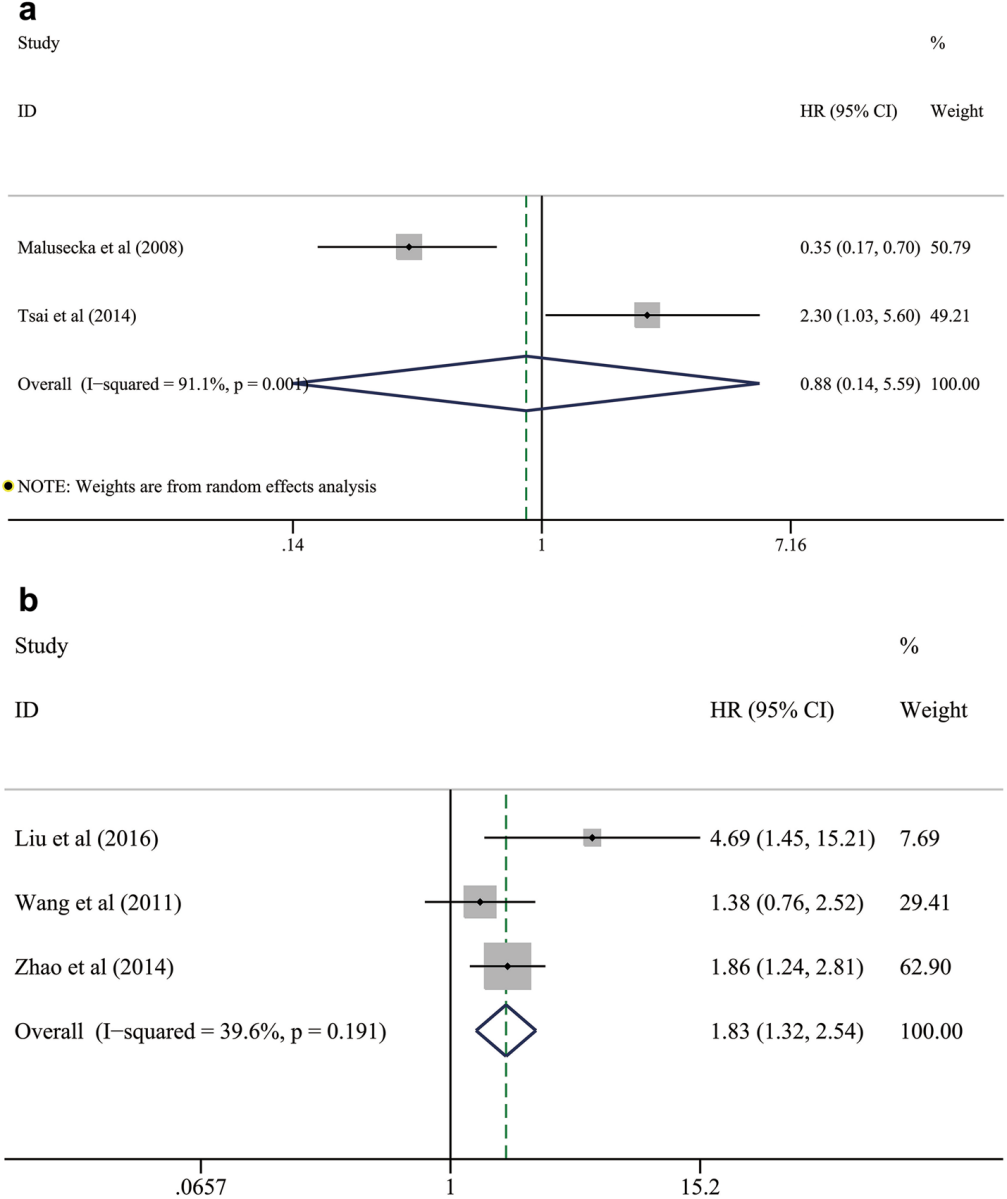
Pooled analyses for assessing the prognostic value of HSP27 expression for **a** 1-year OS and **b** 5-year OS in patients with NSCLC. *HSP27* heat shock protein 27, *NSCLC* non-small cell lung cancer; *OS* overall survival

On the one hand, the pooled HR for 1-year OS based on two included studies (Malusecka et al. 2008; Tsai et al. 2014) was 0.885 (95 % CI 0.140–5.599; *P* = 0.896; I^2^ = 91.5 %, *p* = 0.001; Fig. 4a; Table 3), indicating that there was no significant correlation between HSP27 expression and 1-year OS of NSCLC. On the other hand, the integrated estimates for 5-year OS based on three included studies (Liu et al. 2016; Wang et al. 2011; Zhao et al. 2014a) suggested that HSP27 expression significantly predicted a worse prognosis in patients with NSCLC (HR: 1.832; 95 % CI 1.322–2.538; *P* < 0.001; I^2^ = 39.6 %, *p* = 0.191; Fig. 4b; Table 3). Moreover, further assessment of the association between HSP27 expression and long-term survival outcomes (10-year OS) was given up because of the scarcity of included studies and available results.

**Table 3:** Meta-analyses of the prognostic value of HSP27 expression in patients with NSCLC

Survival outcomes	*N*	No. samples			Heterogeneity (I-squared, *p*)	Model	HR (95 % CI)	*P* value	Conclusion
		Total	PE	NE					
1-year OS	2	140	98	42	91.5 %, 0.001	Random	0.885 (0.140–5.599)	0.896	Not significant
5-year OS	3	334	185	149	39.6 %, 0.191	Fixed	1.832 (1.322–2.538)	<0.001	Significant
10-year OS	Given up because of the scarcity of included studies								

*CI* confidence interval, *HR* hazard ratio, *HSP27* heat shock protein 27, *N* reference count, *NE* negative expression, *NSCLC* non-small cell lung cancer, *OS* overall survival, *PE* positive expression

### Sensitivity analysis

We conducted further sensitivity analysis and additional adjustments in both the evaluations for clinicopathological and prognostic significance of HSP27 expression in NSCLC (the derived forest plots not shown). Finally, very high heterogeneity was only observed between two studies (Malusecka et al. 2008; Tsai et al. 2014) evaluating the relationship between HSP27 expression and 1-year OS (I^2^ = 91.5 %, *p* = 0.001). Both of the primary HR outcomes from these two studies were out of the estimated range by omitting each one study, respectively. Thus, the primary pooled data suggesting no significant correlation between HSP27 expression and 1-year OS might not be reliable enough. The strong robustness of our meta-analyses for other clinicopathological characteristics and prognostic value of HSP27 expression for 5-year OS of NSCLC was confirmed by further sensitivity analysis.

### Publication bias

There was no evidence for potential publication bias within the present meta-analysis. The funnel plots derived from Begg’s test were not showed but their corresponding Egger’s and Begg’s *P* values were outlined in Table 4. Remarkably, we must warn about the poor efficacies of both Begg’s test and Egger’s test when including far less than 20 studies. Although no significant publication bias was revealed in our study, a poor sensitivity of both Begg’s test and Egger’s test should be seriously concerned because of the scarcity of enough included studies.

**Table 4:** Assessments for the potential publication bias within the meta-analysis

Groups of outcomes	*N*	Estimates	Begg’s test (*p* value)	Egger’s test (*p* value)	Publication bias
Clinicopathological characteristics					
Gender (male vs female)	7	OR with 95 % CI	1.0	0.410	Not significant
Age (≥60 years vs <60 years)	3	OR with 95 % CI	1.0	0.645	Not significant
Smoking status (yes vs no)	3	OR with 95 % CI	1.0	0.985	Not significant
Differentiation degree (G3 vs G1 and G2)	8	OR with 95 % CI	0.322	0.120	Not significant
Lymphatic metastasis (yes vs no)	8	OR with 95 % CI	0.322	0.160	Not significant
TNM stage (III/IV vs I/II)	5	OR with 95 % CI	0.806	0.400	Not significant
Histological subtypes (SCC vs AC)	4	OR with 95 % CI	1.0	0.309	Not significant
Tumor size (≥3 cm vs <3 cm)	2	OR with 95 % CI	1.0	Not reported	Not significant
Prognostic significance					
1-year OS	2	HR with 95 % CI	1.0	Not reported	Not significant
5-year OS	3	HR with 95 % CI	1.0	0.564	Not significant

*AC* adenocarcinoma, *CI* confidence interval, *HR* hazard ratio, *OR* odds ratio, *OS* overall survival, *SCC* squamous cell carcinoma

## Discussion

Meta-analysis is a well-designed statistical method integrating the common outcome data from homogeneous studies to settle global conclusions on a pending issue (Zhu et al. 2016; Song et al. 2016; Kubo et al. 2015; Li et al. 2015, 2016a, b, c). To the best of our knowledge, this is the first comprehensive meta-analysis to determine the association between HSP27 expression and major clinicopathological features of NSCLC, and the prognostic roles of HSP27 in NSCLC. On the one hand, our meta-analysis showed that HSP27 expression was significantly associated with worse pathological characteristics of NSCLC, including poor differentiation degree, lymphatic metastasis, advanced TNM stage, SCC and larger tumor size. However, no association was observed between HSP27 expression and other clinical variables, including age, gender and smoking status. On the other hand, positive expression of HSP27 significantly predicted the worse 5-year OS in patients with NSCLC. However, there was no prognostic value of HSP27 expression on 1-year OS of NSCLC based on the pooled analysis. Potential prognostic roles of HSP27 for long-time survival could not be estimated because of the scarcity of the currently available evidences.

HSPs are firstly discovered in 1962 (Sadikovic et al. 2008). The following laboratorial investigations have clarified the universal responses of HSPs to hyperthermia and other physiological stresses in almost all cell types (Vidyasagar et al. 2012; Kaigorodova and Bogatyuk 2014). HSP27 belongs to the small HSP family with a molecular weight about 12–43 kDa (Kim et al. 1998). It is generally regarded as a protein chaperone preventing the irreversible oligomerization and inducing the refolding of damaged proteins (Rogalla et al. 1999). Cytoplasmic HSP27 has some protective effects on the stress conditions, such as oxidative stress and chemical stress. Arrigo et al. (2005) has described the anti-oxidation function of cytoplasmic HSP27 by increasing intracellular glutathione level and decreasing intracellular iron level. Reactive oxygen species are largely controlled by the inhibitions from HSP27. As for chemical stress, many preclinical studies have identified the anti-apoptotic functions of HSP27 achieved by interacting with both mitochondria dependent and independent pathways of apoptosis (Kaigorodova and Bogatyuk 2014). On the basis of these discoveries, Nakashima et al. (2011) recognized the impact of HSP27 phosphorylation status on gemcitabine-induced growth suppression of pancreatic cancer. Hansen et al. (1999) demonstrated that HSP27 could serve as an anti-apoptotic agent during doxorubicin-induced apoptosis of human breast cancer cells, and indicated the inhibition of HSP27 expression in certain breast cancer chemotherapies. However, the latest study reported by Li et al. (2014) showed that the anti-tumor drug bufalin could trigger apoptosis by targeting HSP27 and it might become a novel therapeutic agent for pancreatic cancer. The underlying molecular mechanisms have not been well understood by now and more fundamental researches are required in the future.

HSP27 nuclear localization has been found to regulate the expression of gene controlling mRNA degradation and cell differentiation in erythrocytes (de Thonel et al. 2010; Li et al. 2013). Sedlackova et al. (2011) investigated the corresponding mRNA profiles of HSP27 and attempted to demonstrate the functions of HSP27 as a biomarker for differentiation states. However, a limited sample size may cause negative effects on dissecting the correlation and lead to the observation of no diagnostic or prognostic value. Serum concentration of HSP27 in both chronic obstructive pulmonary disease and NSCLC was firstly studied by a research team from Austria (Zimmermann et al. 2012, 2014; Hacker et al. 2009; Ankersmit et al. 2015). Zimmermann et al. (2012) conducted a multi-institutional case–control study to assess the potentially diagnostic value of HSP27 in NSCLC. Their outcome data suggested that serum HSP27 levels could be considered as an effective tool to discriminate between early and advanced stages of NSCLC, and COPD was the leading risk factor for NSCLC. Such diagnostic roles of circulating HSP27 was further proved in their next study with 40 NSCLC cases and 40 healthy controls by different enzyme-linked immunosorbent assays (Zimmermann et al. 2014). Moreover, Schweiger et al. (2015) from the same Austrian team also argued that HSP27 was highly expressed in the tumor stroma of colorectal cancers (CRCs) and might play crucial roles in the projection of life duration in patients with pulmonary metastasis from CRCs, suggesting its potentially prognostic value in CRCs. It will be of great interest to bring above important discoveries into the realm of clinics to help to present a biomarker that can significantly predict the development of NSCLC (Ankersmit et al. 2015). Because of the limited sample availability in the current studies, a consensus based on a larger number of patients needs to be drawn in the future.

Given above reviews, some clinical reports have attempted to reveal a potentially prognostic significance of HSP27 expression in many cancers, such as esophageal cancer (Xue et al. 2014), ovarian cancer (Zhao et al. 2014b), etc. As for the issue we concerned, both two relevant reviews accomplished by Vidyasagar et al. (2012) and Kaigorodova and Bogatyuk (2014) proposed the possible prognostic roles of HSP27 in NSCLC, but totally contradictory conclusions were finally drawn. It seems that these inconsistent results cannot be well-interpreted and there remains a controversy on the relationship between HSP27 expression and the prognosis of NSCLC. We recognized that the incomplete references describing the prognostic value of HSP27 in NSCLC might largely reduce the accuracy of summarized outcomes. The differences in prognostic indicators could also cause adverse effects on reaching a consensus on the prognostic value of HSP27. Thus, in our meta-analysis, we integrated all of the currently available evidences together to achieve comprehensive and detailed assessments.

Systematic searching four electronic databases made us identify a total of five full-text papers focusing on the prognostic value of HSP27 in NSCLC. The earliest study performed by Malusecka et al. (2008) using multivariate analysis indicated that HSP27 expression could predict the survival of NSCLC patients. Their outcome data revealed a significantly longer survival time in patients with positive HSP27 expression signals although only 1-year OS and 3-year OS were available (*P* = 0.003). Similarly, Tsai et al. (2014) also performed a survival analysis in 64 NSCLC patients and estimated the prognostic significance of HSP27 for 1-year OS. However, a significantly worse prognosis in patients with positive expression of HSP27 was finally observed at the endpoint of follow-up (*P* = 0.04). As for 5-year OS, two retrospective studies were conducted based on a larger sample size. The largest one reported by Zhao et al. (2014a) enrolled 209 NSCLC patients and compared the 5-year OS in patients with positive HSP27 expression and those with negative expression. Finally, a significantly higher survival rate appeared in the cohorts with negative HSP27 expression (55.9 vs 34.5 %, *P* = 0.003). Such predictive value of HSP27 for 5-year OS was also supported by what Liu et al. (2016) reported in their latest study with 22 adenocarcinoma cases. Wang et al. (2011) also carried out a survival analysis among 103 NSCLC cases, although no evidence was finally conducted for a significant correlation between HSP27 expression and 5-year OS. No previously associated studies revealing the predictive significance of HSP27 expression for 10-year OS were reported. We speculate that the very low 10-year OS of NSCLC patients itself may cause the lack of available analysis, especially in some observational studies based on a small number of enrolled patients.

Therefore, we classified the outcome data of these five studies into two groups to evaluate the prognostic values of HSP27 for 1-year OS and 5-year OS, respectively. Finally, high HSP27 expression was found to be significantly associated with the lower 5-year OS, indicating that HSP27 can be a strong biomarker predicting the worse 5-year OS of NSCLC. However, no significant relationship was observed between HSP27 expression and 1-year OS, and a remarkable heterogeneity (I^2^ = 91.5 %, *p* = 0.001) existed between studies. Our findings suggested two possible reasons causing such huge differences between 1-year OS group and 5-year OS group: (I) the larger sample size might contribute to figure out the prognostic value of HSP27 for 5-year OS rather than 1-year OS. The derived data might show stable changes with the increase of sample size; (II) the potential impact of different adjuvant therapies on NSCLC patients might cause a variety of survival outcomes during different follow-up periods. There was evidence suggesting that HSP27 overexpression in NSCLC tissues might significantly induce patients’ tolerance to chemotherapeutic agents if treated for a long time (Ciocca and Calderwood 2005). Thus, the prognosis of NSCLC might be influenced by the different responses to adjuvant therapies with the prolongation of follow-up period. The poor responses to treatment could cause adverse effects on the OS of NSCLC (Tsai et al. 2014). Although many possible molecular mechanisms have been recently proposed, the correct one still needs to be clarified by further studies.

In addition, we compared the HSP27 expression in patients with different clinicopathological characteristics of NSCLC. Their pooled ORs indicated that HSP27 expression might be significantly associated with the worse directions for major pathological characteristics of NSCLC, including differentiation degree, lymphatic metastasis, TNM stage, histological subtypes and tumor size. These pathological parameters were universally considered to responsible for poor prognosis of NSCLC. Therefore, we speculated that such relationship between positive expression of HSP27 and clinicopathological characteristics might be able to interpret the prognostic value of HSP27 for 5-year OS of NSCLC to some extent, although its validity was still merited to be verified by further studies.

## Limitations

Finally, several limitations should be seriously considered for accurate interpretations of our pooled analyses. First, the estimations on the relationship between HSP27 expression and clinicopathological characteristics was only based on 714 NSCLC cases from eight retrospective observational studies. Similarly, the pooled analysis assessing the prognostic roles of HSP27 was only based on 474 NSCLC cases from five retrospective observational studies. It should be the leading limitation for our meta-analysis with including so small number of the current evidences. The validity and accuracy of all the integrated estimates should be seriously considered in clinical practices. Second, the scarcity of a uniform cut-off definition for positive HSP27 expression might be the major confounding factor affecting the validity of this meta-analysis. Moreover, different detecting methods supplied by the researchers might cause deviation from the actual value to some extent. Third, a significant heterogeneity was revealed between studies evaluating the prognostic value of HSP27 for 1-year OS. But we could not identify its possible origins by further sensitivity analysis. We speculated that the lack of available studies publishing the 1-year survival outcomes might be the main reason for such a high heterogeneity. We hope we can collect more convincing outcome data by pooling more eligible literatures in the future. Lastly, we found that the majority of enrolled samples come from China. Clinicians should judiciously evaluate the generality of the combined estimates in the clinical settings of their own nations.

## Conclusions

In conclusion, our meta-analysis indicates that HSP27 expression can be an effective biomarker for predicting the poor clinicopathological characteristics of NSCLC, including the differentiation degree, lymphatic metastasis, TNM stage, histological subtypes and tumor size. Meanwhile, HSP27 expression is also a strong predictor for the poor 5-year OS in patients with NSCLC. Because of some inherent restrictions in our meta-analysis, more large-scale studies are urgently required to further confirm these findings, and provide more comprehensive evidences to assess the impact of HSP27 up-regulation on short-term and long-term survival outcomes of NSCLC in the future.

## Additional files

10.1186/s40064-016-2827-8 PRISMA 2009 checklist.
10.1186/s40064-016-2827-8 Summary of the electronic literature retrieval.

## References

[CR1] Alberg AJ, Brock MV, Ford JG, Samet JM, Spivack SD (2013). Epidemiology of lung cancer: diagnosis and management of lung cancer, 3rd ed: American College of Chest Physicians evidence-based clinical practice guidelines. Chest.

[CR2] Ankersmit HJ, Lambers C, Zimmermann M, Hacker S, Moser B (2015). Serendipity and technical considerations for the measurement of serum heat shock protein HSP27 in patients with COPD and lung cancer. Cell Stress Chaperones.

[CR3] Arrigo AP, Virot S, Chaufour S, Firdaus W, Kretz-Remy C, Diaz-Latoud C (2005). Hsp27 consolidates intracellular redox homeostasis by upholding glutathione in its reduced form and by decreasing iron intracellular levels. Antioxid Redox Signal.

[CR4] Begg CB, Mazumdar M (1994). Operating characteristics of a rank correlation test for publication bias. Biometrics.

[CR5] Ciocca DR, Calderwood SK (2005). Heat shock proteins in cancer: diagnostic, prognostic, predictive, and treatment implications. Cell Stress Chaperones.

[CR6] de Thonel A, Vandekerckhove J, Lanneau D, Selvakumar S, Courtois G, Hazoume A, Brunet M, Maurel S, Hammann A, Ribeil JA, Zermati Y, Gabet AS, Boyes J, Solary E, Hermine O, Garrido C (2010). HSP27 controls GATA-1 protein level during erythroid cell differentiation. Blood.

[CR7] Efird JT, Landrine H, Shiue KY, O’Neal WT, Podder T, Rosenman JG, Biswas T (2014). Race, insurance type, and stage of presentation among lung cancer patients. Springerplus.

[CR8] Ferlay J, Shin HR, Bray F, Forman D, Mathers C, Parkin DM (2010). Estimates of worldwide burden of cancer in 2008: GLOBOCAN 2008. Int J Cancer.

[CR9] Guo H, Bai Y, Xu P, Hu ZB, Liu L, Wang F, Jin GF, Wang F, Deng QF, Tu YX, Feng MH, Lu DR, Shen HB, Wu TC (2010). Functional promoter-1271G > C variant of HSPB1 predicts lung cancer risk and survival. J Clin Oncol.

[CR10] Hacker S, Lambers C, Hoetzenecker K, Pollreisz A, Aigner C, Lichtenauer M, Mangold A, Niederpold T, Zimmermann M, Taghavi S, Klepetko W, Ankersmit HJ (2009). Elevated HSP27, HSP70 and HSP90 alpha in chronic obstructive pulmonary disease: markers for immune activation and tissue destruction. Clin Lab.

[CR11] Hansen RK, Parra I, Lemieux P, Oesterreich S, Hilsenbeck SG, Fuqua SAW (1999). Hsp27 overexpression inhibits doxorubicin-induced apoptosis in human breast cancer cells. Breast Cancer Res Tr.

[CR12] Haslbeck M, Franzmann T, Weinfurtner D, Buchner J (2005). Some like it hot: the structure and function of small heat-shock proteins. Nat Struct Mol Biol.

[CR13] Higgins JP, Thompson SG (2002). Quantifying heterogeneity in a meta-analysis. Stat Med.

[CR14] Higgins JP, Thompson SG, Deeks JJ, Altman DG (2003). Measuring inconsistency in meta-analyses. BMJ.

[CR15] Hoffman PC, Mauer AM, Vokes EE (2000). Lung cancer. Lancet.

[CR16] Huang Q, Zu Y, Fu X, Wu T (2005). Expression of heat shock protein 70 and 27 in non-small cell lung cancer and its clinical significance. J Huazhong Univ Sci Technolog Med Sci.

[CR17] Jemal A, Bray F, Center MM, Ferlay J, Ward E, Forman D (2011). Global cancer statistics. CA Cancer J Clin.

[CR18] Kaigorodova EV, Bogatyuk MV (2014). Heat shock proteins as prognostic markers of cancer. Curr Cancer Drug Tar.

[CR19] Kim KK, Kim R, Kim SH (1998). Crystal structure of a small heat-shock protein. Nature.

[CR20] Kubo T, Shimose S, Fujimori J, Furuta T, Ochi M (2015). Prognostic value of SS18-SSX fusion type in synovial sarcoma; systematic review and meta-analysis. Springerplus.

[CR21] Li R, Fan X, Zhong H, Chen G, Feng J, Han B (2005). Clinical significance and expression of cyclooxygenase-2, vascular endothelial growth factor, heat shock protein 27 and P53 in human non-small cell lung carcinoma. Shanghai Med.

[CR22] Li ML, Defren J, Brewer G (2013). Hsp27 and F-box protein beta-TrCP promote degradation of mRNA decay factor AUF1. Mol Cell Biol.

[CR23] Li M, Yu X, Guo H, Sun L, Wang A, Liu Q, Wang X, Li J (2014). Bufalin exerts antitumor effects by inducing cell cycle arrest and triggering apoptosis in pancreatic cancer cells. Tumour Biol.

[CR24] Li YJ, Dai YL, Zhang WB, Li SJ, Tu CQ (2015). Clinicopathological and prognostic significance of chemokine receptor CXCR4 in patients with bone and soft tissue sarcoma: a meta-analysis. Clin Exp Med.

[CR25] Li S, Fan J, Liu J, Zhou J, Ren Y, Shen C, Che G (2016). Neoadjuvant therapy and risk of bronchopleural fistula after lung cancer surgery: a systematic meta-analysis of 14 912 patients. Jpn J Clin Oncol.

[CR26] Li S, Lai Y, Fan J, Shen C, Che G (2016). Clinicopathological and prognostic significance of Nestin expression in patients with non-small cell lung cancer: a systematic review and meta-analysis. Clin Exp Med.

[CR27] Li SJ, Fan J, Zhou J, Ren YT, Shen C, Che GW (2016). Diabetes mellitus and risk of bronchopleural fistula after pulmonary resections: a meta-analysis. Ann Thorac Surg.

[CR28] Liu CL, Chen SF, Wu MZ, Jao SW, Lin YS, Yang CY, Lee TY, Wen LW, Lan GL, Nieh S (2016). The molecular and clinical verification of therapeutic resistance via the p38 MAPK-Hsp27 axis in lung cancer. Oncotarget.

[CR29] Malusecka E, Krzyzowska-Gruca S, Gawrychowski J, Fiszer-Kierzkowska A, Kolosza Z, Krawczyk Z (2008). Stress proteins HSP27 and HSP70i predict survival in non-small cell lung carcinoma. Anticancer Res.

[CR30] Moher D, Liberati A, Tetzlaff J, Altman DG, Group P (2009) Preferred reporting items for systematic reviews and meta-analyses: the PRISMA statement. BMJ 339:b2535. doi:10.1136/bmj.b2535 PMC3090117 21603045

[CR31] Nakashima M, Adachi S, Yasuda I, Yamauchi T, Kawaguchi J, Itani M, Yoshioka T, Matsushima-Nishiwaki R, Hirose Y, Kozawa O, Moriwaki H (2011). Phosphorylation status of heat shock protein 27 plays a key role in gemcitabine-induced apoptosis of pancreatic cancer cells. Cancer Lett.

[CR32] Norton JA, Weinberger PM, Waller JL, Merkley MA, Jackson LL, Dynan WS (2010). Significance of HSPB1 expression in head and neck squamous cell carcinoma: a meta-analysis of published literatures. Laryngoscope.

[CR33] Parmar MKB, Torri V, Stewart L (1998). Extracting summary statistics to perform meta-analyses of the published literature for survival endpoints. Stat Med.

[CR34] Rogalla T, Ehrnsperger M, Preville X, Kotlyarov A, Lutsch G, Ducasse C, Paul C, Wieske M, Arrigo AP, Buchner J, Gaestel M (1999). Regulation of Hsp27 oligomerization, chaperone function, and protective activity against oxidative stress tumor necrosis factor alpha by phosphorylation. J Biol Chem.

[CR35] Sadikovic B, Al-Romaih K, Squire JA, Zielenska M (2008). Cause and consequences of genetic and epigenetic alterations in human cancer. Curr Genomics.

[CR36] Schweiger T, Nikolowsky C, Starlinger P, Traxler D, Zimmermann M, Birner P, Hegedus B, Dome B, Bergmann M, Mildner M, Klepetko W, Hoetzenecker K, Ankersmit HJ (2015). Stromal expression of heat-shock protein 27 is associated with worse clinical outcome in patients with colorectal cancer lung metastases. PLoS ONE.

[CR37] Sedlackova L, Spacek M, Holler E, Imryskova Z, Hromadnikova I (2011). Heat-shock protein expression in leukemia. Tumour Biol.

[CR38] Song W, Wang K, Zhang RJ, Dai QX, Zou SB (2016). The enhanced recovery after surgery (ERAS) program in liver surgery: a meta-analysis of randomized controlled trials. Springerplus.

[CR39] Stang A (2010). Critical evaluation of the Newcastle-Ottawa scale for the assessment of the quality of nonrandomized studies in meta-analyses. Eur J Epidemiol.

[CR40] Tierney JF, Stewart LA, Ghersi D, Burdett S, Sydes MR (2007). Practical methods for incorporating summary time-to-event data into meta-analysis. Trials.

[CR41] Tsai JR, Liu PL, Chen YH, Chou SH, Yang MC, Cheng YJ, Hwang JJ, Yin WH, Chong IW (2014). Ginkgo biloba extract decreases non-small cell lung cancer cell migration by downregulating metastasis-associated factor heat-shock protein 27. PLoS ONE.

[CR42] Vidyasagar A, Wilson NA, Djamali A (2012). Heat shock protein 27 (HSP27): biomarker of disease and therapeutic target. Fibrogenesis Tissue Repair.

[CR43] Wang W, Xu X, Wang W, Shao W, Li L, Yin W, Xiu L, Mo M, Zhao J, He Q, He J (2011). The expression and clinical significance of CLIC1 and HSP27 in lung adenocarcinoma. Tumour Biol.

[CR44] Wu YL, Lee JS, Thongprasert S, Yu CJ, Zhang L, Ladrera G, Srimuninnimit V, Sriuranpong V, Sandoval-Tan J, Zhu Y, Liao M, Zhou C, Pan H, Lee V, Chen YM, Sun Y, Margono B, Fuerte F, Chang GC, Seetalarom K, Wang J, Cheng A, Syahruddin E, Qian X, Ho J, Kurnianda J, Liu HE, Jin K, Truman M, Bara I, Mok T (2013). Intercalated combination of chemotherapy and erlotinib for patients with advanced stage non-small-cell lung cancer (FASTACT-2): a randomised, double-blind trial. Lancet Oncol.

[CR45] Xue L, Yang L, Jin ZA, Gao F, Kang JQ, Xu GH, Liu B, Li H, Wang XJ, Liu LJ, Wang BL, Liang SH, Ding J (2014). Increased expression of HSP27 inhibits invasion and metastasis in human esophageal squamous cell carcinoma. Tumour Biol.

[CR46] Yao HX, Zhang ZQ, Xiao ZQ, Chen YH, Li C, Zhang PF, Li MX, Liu YF, Guan YJ, Yu YH, Chen ZC (2009). Identification of metastasis associated proteins in human lung squamous carcinoma using two-dimensional difference gel electrophoresis and laser capture microdissection. Lung Cancer.

[CR47] Zhao Y, Xie W, Feng M, Yu J, Duan L, Wu H (2009). Expressions of HSP27 in lung carcinoma and its clinical implication. China J Mod Med.

[CR48] Zhao GY, Ding JY, Gu J, Lu CL, Lin ZW, Guo J, Ge D (2014). The Overexpression of 14-3-3 zeta and Hsp27 promotes non-small cell lung cancer progression. Cancer Am Cancer Soc.

[CR49] Zhao M, Ding JX, Zeng K, Zhao J, Shen F, Yin YX, Chen Q (2014). Heat shock protein 27: a potential biomarker of peritoneal metastasis in epithelial ovarian cancer?. Tumour Biol.

[CR50] Zhu L, Chen S, Ma S, Zhang S (2016). Glasgow prognostic score predicts prognosis of non-small cell lung cancer: a meta-analysis. Springerplus.

[CR51] Zimmermann M, Nickl S, Lambers C, Hacker S, Mitterbauer A, Hoetzenecker K, Rozsas A, Ostoros G, Laszlo V, Hofbauer H, Renyi-Vamos F, Klepetko W, Dome B, Ankersmit HJ (2012). Discrimination of clinical stages in non-small cell lung cancer patients by serum HSP27 and HSP70: a multi-institutional case-control study. Clinica Chimica Acta.

[CR52] Zimmermann M, Mueller T, Dieplinger B, Bekos C, Beer L, Hofbauer H, Dome B, Ankersmit HJ (2014). Circulating heat shock protein 27 as a biomarker for the differentiation of patients with lung cancer and healthy controls–a clinical comparison of different enzyme linked immunosorbent assays. Clin Lab.

[CR53] Zu Y, Huang Q (2005). Expression and clinical significance of HSP70 and HSP27 in non-small cell lung cancer. J Clin Surg.

