# Supplementary material for: Clinicopathological and prognostic significance of heat shock protein 27 (HSP27) expression in non-small cell lung cancer: a systematic review and meta-analysis
Source: Springerplus. 2016 Jul 25;5(1):1165. doi: 10.1186/s40064-016-2827-8 (PMC4960090; doi:10.1186/s40064-016-2827-8)
Supplement: Supplementary file 2 — 10.1186/s40064-016-2827-8 Summary of the electronic literature retrieval. [file 40064_2016_2827_MOESM2_ESM.doc]

***Additional file 2***

***Summary of the electronic literature retrieval***

PubMed search strategy

| **Searches** | **Search details** | **Items found** |
| --- | --- | --- |
| **#1** | **Search** (((("hsp27 heat-shock proteins"[MeSH Terms] OR ("hsp27"[All Fields] AND "heat-shock"[All Fields] AND "proteins"[All Fields]) OR "hsp27 heat-shock proteins"[All Fields] OR "heat shock protein 27"[All Fields]) OR (("heat-shock proteins"[MeSH Terms] OR ("heat-shock"[All Fields] AND "proteins"[All Fields]) OR "heat-shock proteins"[All Fields] OR ("heat"[All Fields] AND "shock"[All Fields] AND "protein"[All Fields]) OR "heat shock protein"[All Fields]) AND B1[All Fields])) OR ("hsp27 heat-shock proteins"[MeSH Terms] OR ("hsp27"[All Fields] AND "heat-shock"[All Fields] AND "proteins"[All Fields]) OR "hsp27 heat-shock proteins"[All Fields] OR "hsp27"[All Fields])) OR HSPB1[All Fields]) AND ("lung neoplasms"[MeSH Terms] OR ("lung"[All Fields] AND "neoplasms"[All Fields]) OR "lung neoplasms"[All Fields] OR ("lung"[All Fields] AND "cancer"[All Fields]) OR "lung cancer"[All Fields]) | **111** |
| **#2** | **Search** (((("hsp27 heat-shock proteins"[MeSH Terms] OR ("hsp27"[All Fields] AND "heat-shock"[All Fields] AND "proteins"[All Fields]) OR "hsp27 heat-shock proteins"[All Fields] OR "heat shock protein 27"[All Fields]) OR (("heat-shock proteins"[MeSH Terms] OR ("heat-shock"[All Fields] AND "proteins"[All Fields]) OR "heat-shock proteins"[All Fields] OR ("heat"[All Fields] AND "shock"[All Fields] AND "protein"[All Fields]) OR "heat shock protein"[All Fields]) AND B1[All Fields])) OR ("hsp27 heat-shock proteins"[MeSH Terms] OR ("hsp27"[All Fields] AND "heat-shock"[All Fields] AND "proteins"[All Fields]) OR "hsp27 heat-shock proteins"[All Fields] OR "hsp27"[All Fields])) OR HSPB1[All Fields]) AND (("lung"[MeSH Terms] OR "lung"[All Fields]) AND ("carcinoma"[MeSH Terms] OR "carcinoma"[All Fields])) | **55** |
| **#3** | **Search** (((("hsp27 heat-shock proteins"[MeSH Terms] OR ("hsp27"[All Fields] AND "heat-shock"[All Fields] AND "proteins"[All Fields]) OR "hsp27 heat-shock proteins"[All Fields] OR "heat shock protein 27"[All Fields]) OR (("heat-shock proteins"[MeSH Terms] OR ("heat-shock"[All Fields] AND "proteins"[All Fields]) OR "heat-shock proteins"[All Fields] OR ("heat"[All Fields] AND "shock"[All Fields] AND "protein"[All Fields]) OR "heat shock protein"[All Fields]) AND B1[All Fields])) OR ("hsp27 heat-shock proteins"[MeSH Terms] OR ("hsp27"[All Fields] AND "heat-shock"[All Fields] AND "proteins"[All Fields]) OR "hsp27 heat-shock proteins"[All Fields] OR "hsp27"[All Fields])) OR HSPB1[All Fields]) AND ("lung neoplasms"[MeSH Terms] OR ("lung"[All Fields] AND "neoplasms"[All Fields]) OR "lung neoplasms"[All Fields] OR ("lung"[All Fields] AND "neoplasm"[All Fields]) OR "lung neoplasm"[All Fields]) | **114** |

***EMBASE (via Ovid interface) search strategy***

| **Searches** | **Search details** | **Items found** |
| --- | --- | --- |
| **#1** | **Search** ((heat shock protein 27 or heat shock protein B1 or HSP27 or HSPB1) and lung cancer).af. | **115** |
| **#2** | **Search** ((heat shock protein 27 or heat shock protein B1 or HSP27 or HSPB1) and lung carcinoma).af. | **28** |
| **#3** | **Search** ((heat shock protein 27 or heat shock protein B1 or HSP27 or HSPB1) and lung neoplasm).af. | **0** |

***The Web of Science (via campus network of Sichuan University)*** search strategy

| **Searches** | **Search details** | **Items found** |
| --- | --- | --- |
| **#1** | **Search** TS=((heat shock protein 27 OR heat shock protein B1 OR HSP27 OR HSPB1) AND lung cancer) | **245** |
| **#2** | **Search** TS=((heat shock protein 27 OR heat shock protein B1 OR HSP27 OR HSPB1) AND lung carcinoma) | **145** |
| **#3** | **Search** TS=((heat shock protein 27 OR heat shock protein B1 OR HSP27 OR HSPB1) AND lung neoplasm) | **126** |

* ***The complete details of search strings in CNKI database are not given because of the Chinese words used during the literature retrieval.***
